# Advances in cell therapy for orthopedic diseases: bridging immune modulation and regeneration

**DOI:** 10.3389/fimmu.2025.1567640

**Published:** 2025-04-10

**Authors:** Jing Wang, Shenghao Xu, Bo Chen, Yanguo Qin

**Affiliations:** ^1^ Department of Orthopedics, The Second Hospital of Jilin University, Changchun, Jilin, China; ^2^ Joint International Research Laboratory of Ageing Active Strategy and Bionic Health in Northeast Asia of Ministry of Education, Jilin University, Changchun, Jilin, China

**Keywords:** cell therapy, orthopedic diseases, immunotherapy, MSCs, iPSCs

## Abstract

Orthopedic diseases pose significant challenges to public health due to their high prevalence, debilitating effects, and limited treatment options. Additionally, orthopedic tumors, such as osteosarcoma, chondrosarcoma, and Ewing sarcoma, further complicate the treatment landscape. Current therapies, including pharmacological treatments and joint replacement, address symptoms but fail to promote true tissue regeneration. Cell-based therapies, which have shown successful clinical results in cancers and other diseases, have emerged as a promising solution to repair damaged tissues and restore function in orthopedic diseases and tumors. This review discusses the advances and potential application of cell therapy for orthopedic diseases, with a particular focus on osteoarthritis, bone fractures, cartilage degeneration, and the treatment of orthopedic tumors. We explore the potential of mesenchymal stromal cells (MSCs), chondrocyte transplantation, engineered immune cells and induced pluripotent stem cells to enhance tissue regeneration by modulating the immune response and addressing inflammation. Ultimately, the integration of cutting-edge cell therapy, immune modulation, and molecular targeting strategies could revolutionize the treatment of orthopedic diseases and tumors, providing hope for patients seeking long-term solutions to debilitating conditions.

## 1 Introduction

### 1.1 Orthopedic diseases and the need for regenerative treatments

Orthopedic diseases, including osteoarthritis (OA), bone fractures, cartilage degeneration, and orthopedic tumors, are among the most prevalent conditions worldwide (1–3). These diseases significantly impact the quality of life of millions, particularly in aging populations. Osteoarthritis alone affects over 500 million people globally, with its incidence expected to rise as the population ages (1). Bone cancers such as osteosarcoma, chondrosarcoma, and Ewing sarcoma, although rarer, present significant clinical challenges in both diagnosis and treatment, requiring more specialized care. These diseases are characterized by the progressive deterioration of joints, cartilage, and bone, which leads to pain, reduced mobility, and impaired function (**Table 1**). Orthopedic tumors, though often less common, complicate treatment regimens with the need for aggressive therapies that include surgery, chemotherapy, and radiation. Current pharmacological treatments for pain management in degenerative joint diseases include nonsteroidal anti-inflammatory drugs (NSAIDs), opioids, corticosteroids, and disease-modifying osteoarthritis drugs (DMOADs). These agents aim to reduce pain and inflammation, although they do not address the underlying degeneration of joint tissues. While conventional treatments like pharmacological management and joint replacement surgeries help address symptoms, they fail to promote true tissue regeneration (4, 5). These treatments can be effective in alleviating pain and restoring some joint function, but they do not address the underlying tissue loss or repair the natural anatomy of the affected areas.

**Table 1:** Pathological traits, subtypes, and grading criteria of key diseases affecting joints and bones.

Disease	Pathological Traits	Subtypes/Grading Criteria
Osteoarthritis (OA)	- Degeneration of articular cartilage	- Early: Cartilage thinning
	- Bone remodeling	- Moderate: Cartilage loss with osteophyte formation
	- Inflammation of synovial membrane	- Severe: Joint deformity and loss of function
Rheumatoid Arthritis (RA)	- Chronic inflammation of synovial membranes	- Early: Synovitis
	- Pannus formation	- Moderate: Joint destruction
	- Joint erosion and deformity	- Severe: Deformity and ankylosis
Bone Tumors	- Uncontrolled growth of bone or soft tissue	- Benign: Osteochondroma, Enchondroma
	- Can be benign or malignant	- Malignant: Osteosarcoma, Ewing’s sarcoma, Chondrosarcoma
	- Pain, swelling, and functional impairment	

Regenerative medicine, particularly cell-based therapies, has gained attention as a promising alternative to traditional treatments. By harnessing the body’s own regenerative potential, these therapies aim to repair or regenerate damaged tissues and restore function (6, 7). This approach is being explored not only for degenerative diseases like osteoarthritis but also for orthopedic tumors, where regenerative treatments may aid in rebuilding bone tissue after tumor excision or radiation (8). However, despite significant advances, challenges remain in optimizing the efficacy and long-term outcomes of cell therapies in orthopedic diseases. The inability to fully regenerate damaged tissues, control inflammation, and address immune responses within the local tissue microenvironment remains a significant barrier to the successful application of these therapies (9, 10).

### 1.2 Engineered cell therapies

Cell therapy has emerged as a promising solution to address the limitations of conventional treatments for orthopedic diseases, including degenerative conditions and tumors. Mesenchymal stromal cells (MSCs), which have the ability to differentiate into multiple cell types, such as chondrocytes, osteoblasts, and adipocytes, are a key focus of research in this field (11, 12). MSCs can be sourced from various tissues, such as bone marrow, adipose tissue, and synovial fluid, and have shown potential in repairing cartilage defects, promoting bone healing, and reducing inflammation (10, 13, 14). Clinical studies have demonstrated the safety and feasibility of MSC-based therapies for conditions like osteoarthritis, with improvements in pain reduction and function observed in many cases (15). For orthopedic tumors, MSCs may also play a role in regenerating bone and cartilage after tumor resection. MSC-based treatments have been explored in preclinical models for osteosarcoma and other bone cancers, where they may assist in filling bone defects left after surgery and aid in the regeneration of normal bone tissue (16). Another promising approach is chondrocyte transplantation, where autologous chondrocytes are harvested, expanded, and re-implanted into damaged cartilage (17, 18). It has shown success in treating focal cartilage defects, although challenges remain in maintaining the functional integrity of the graft long-term (19). Furthermore, the engineering of immune cells, such as T-cells or macrophages, to enhance their regenerative potential and modulate the inflammatory response within the orthopedic disease microenvironment represents an exciting avenue for research (20). In orthopedic tumors, engineered immune cells also help in targeting residual tumor cells or modulating the immune response to prevent relapse (21). The key to success in these therapies lies in overcoming the inflammatory environment that characterizes many orthopedic diseases and finding ways to enhance tissue regeneration while preventing immune rejection (22, 23). Additionally, induced pluripotent stem cells (iPSCs) have gained attention in orthopedic disease therapy due to their unique ability to differentiate into any cell type, including chondrocytes and osteoblasts, providing the potential for autologous tissue repair. Although iPSCs offer significant promise, challenges in controlling differentiation and preventing tumorigenesis remain (24).

### 1.3 Key molecular pathways modulate the cell therapy effect in orthopedic diseases

A critical factor in the success of cell therapies for orthopedic diseases is the modulation of the immune response and the regulation of local inflammation (25, 26). The immune microenvironment of tissues affected by osteoarthritis, bone fractures, and tumors is often characterized by a pro-inflammatory state that hinders healing and tissue regeneration (27). Toll-like receptors (TLRs), a family of pattern recognition receptors that play a central role in innate immunity, are key regulators of this inflammatory response (28, 29). TLRs are expressed on various cells, including chondrocytes, macrophages, synoviocytes, and even cancer cells, and their activation leads to the production of pro-inflammatory cytokines and chemokines, contributing to cartilage degradation, bone resorption, and tumor progression (30, 31). Among the TLRs, TLR3 has emerged as a particularly interesting target in orthopedic disease therapy (32–34). TLR3 is primarily activated by double-stranded RNA, a pathogen-associated molecular pattern typically associated with viral infections (35). Upon activation, TLR3 initiates a signaling cascade involving the transcription factors NF-κB and interferon regulatory factor 3 (IRF3), which leads to the production of pro-inflammatory cytokines and type I interferons (36, 37). In orthopedic diseases and tumors, TLR3 plays a dual role by modulating the immune response and influencing tissue repair processes. Recent studies suggest that TLR3 signaling may be involved in the regulation of cartilage degeneration, bone remodeling, and immune responses to bone tumors. TLR3 activation promotes joint degeneration in osteoarthrosis (34).

## 2 Advances in cell-based therapies for orthopedic diseases

**Figure 1:**
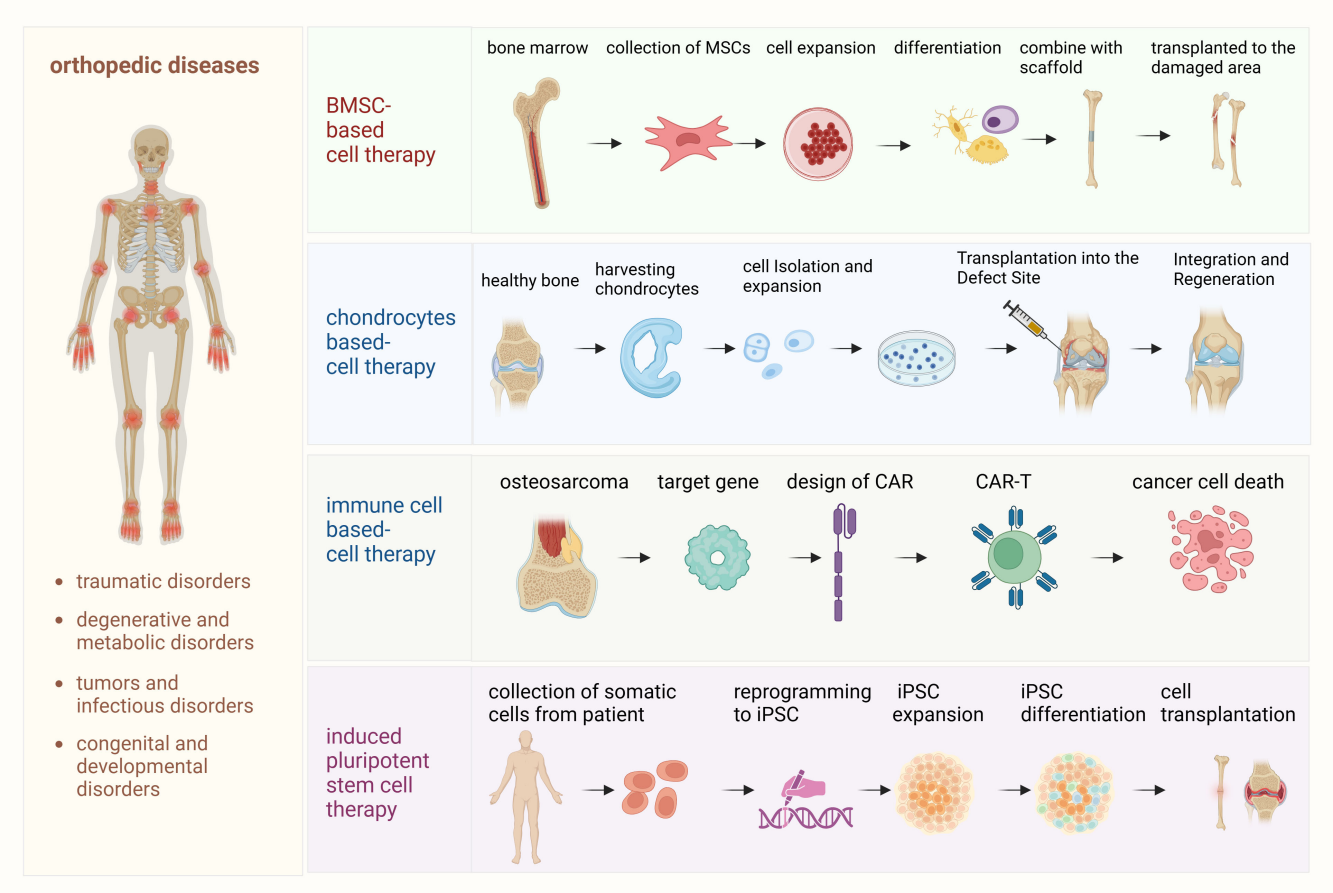
Detailed processes of cell-based therapies for orthopedic diseases. This diagram outlines the step-by-step processes involved in various cell-based therapies used for the treatment of orthopedic diseases. (1) Mesenchymal stem cell (MSC) therapy: This process begins with the isolation of MSCs from bone marrow or adipose tissue. The MSCs are then expanded in culture and differentiated into osteoblasts, chondrocytes, or other cell types, depending on the specific tissue regeneration needed. These differentiated cells are transplanted into the damaged bone or cartilage to promote healing and regeneration. (2) Chondrocyte therapy: In this process, autologous chondrocytes are harvested from the patient’s own cartilage, expanded *in vitro*, and then re-implanted into the damaged cartilage area. This therapy is designed to repair cartilage defects, particularly in cases of osteoarthritis or traumatic injuries. (3) Immune cell therapy (osteosarcoma and CAR-T therapy): This section illustrates the use of chimeric antigen receptor T (CAR-T) cell therapy specifically for osteosarcoma treatment. Patient-derived T cells are engineered to express CARs targeting tumor antigens on osteosarcoma cells. These engineered CAR-T cells are expanded and infused back into the patient, where they recognize and attack tumor cells, modulating the immune response and potentially improving outcomes for patients with osteosarcoma. (4) Induced pluripotent stem cell (iPSC) therapy: iPSCs are generated by reprogramming somatic cells, such as skin fibroblasts, back into pluripotent cells. These iPSCs are then differentiated into osteoblasts or chondrocytes to regenerate bone and cartilage tissues, offering a potential therapeutic approach for a wide range of orthopedic disorders.

Cell-based therapies have shown considerable promise in the field of regenerative medicine, offering potential solutions for treating orthopedic diseases, such as osteoarthritis, cartilage degeneration, bone fractures, and even orthopedic tumors. These therapies aim to repair, regenerate, or replace damaged tissues by harnessing the regenerative properties of different cell types. This section explores several key approaches, including MSC therapy, chondrocyte-based approaches, engineered immune cell therapies and iPSCs, each of which presents unique benefits and challenges (**Figure 1**).

### 2.1 Mesenchymal stroma cell therapy

In this review, the term mesenchymal stromal cells (MSCs) is used in accordance with the International Society for Cell and Gene Therapy (ISCT) nomenclature guidelines (38). MSCs are a population of cells with immunomodulatory and secretory functions, as opposed to ‘mesenchymal stem cells,’ which are defined by their demonstrated self-renewal and multilineage differentiation capabilities. Key markers used to identify MSCs, such as CD73, CD90, and CD105, are essential for characterizing MSCs in regenerative therapies. These markers help define the identity and functional properties of MSCs, ensuring their purity and effectiveness in clinical applications.

MSCs are a promising cell type for regenerative therapies due to their ability to differentiate into various mesodermal cell types, including chondrocytes, osteoblasts, and adipocytes. MSCs are primarily derived from adult tissues, such as bone marrow, adipose tissue, and synovial fluid, and can also be sourced from umbilical cord blood or placenta. Their multipotent differentiation capacity, coupled with their immunomodulatory properties, makes them an ideal candidate for treating orthopedic diseases. MSCs exerts therapeutic effects via paracrine signaling (39–41). When MSCs are transplanted into damaged tissues, they secrete a wide range of bioactive molecules, including growth factors, cytokines, and extracellular matrix components, that help modulate inflammation, stimulate tissue repair, and promote regeneration (42, 43). In osteoarthritis, for example, MSCs secrete anti-inflammatory cytokines such as IL-10 and TGF-β, which help to counteract the inflammatory mediators that contribute to cartilage degradation (44). Moreover, MSCs stimulate the proliferation and differentiation of endogenous progenitor cells, thereby enhancing tissue regeneration. In addition to their regenerative capabilities, MSCs are also capable of modulating the immune response. MSCs interact with various immune cells, such as T cells, B cells, and macrophages, to suppress inflammatory responses and reduce immune-mediated damage in tissues (12, 45, 46). This immunomodulatory effect is particularly important in autoimmune and inflammatory diseases like osteoarthritis, where chronic inflammation is a key driver of disease progression (47, 48). For example, bone marrow MSC (BMSC)-derived exosomes prevent osteoarthritis by regulating synovial macrophage polarization, promoting macrophages from pro-inflammatory (M1-like) phenotype to anti-inflammatory (M2-like) phenotype (39).

MSC-based therapies for orthopedic diseases have shown promising results in preclinical studies and early-phase clinical trials. MSCs are being investigated as a potential therapy for OA, with clinical trials in phase I/II showing pain relief and functional improvement (49, 50). However, challenges like low cell survival in inflammatory joints and limited therapeutic duration remain. Two clinical studies highlight the importance of stem cell delivery sites (44, 51). In one study, bilateral knee OA patients received BMSCs either via intra-articular (I.A.) injection or directly to subchondral bone lesions. After 15 years, 70% of knees in the I.A. group required total knee arthroplasty (TKA), compared to only 20% in the subchondral group, emphasizing the role of BML (bone marrow lesion)-targeted therapy in preventing OA progression (51). Another study on 140 late-stage OA patients compared subchondral BMSC delivery to TKA (44). Both groups had similar knee scores (~80) and TKA rates (~1%/year) after 15 years, suggesting BML regeneration delay knee replacement by over a decade. These findings underscore the potential of BMSC therapy and the critical role of subchondral bone in OA treatment (52).

For bone fractures, MSCs have been used to promote bone healing, particularly in cases of non-union fractures or those associated with large bone defects (53, 54). Hypoxic MSC-derived exosomes promote bone fracture healing by the transfer of miR-126 (40). Moreover, in orthopedic tumors, MSCs have shown potential for assisting in the regeneration of bone tissue after tumor excision or radiation, particularly in bone cancers like osteosarcoma. Osteosarcoma is a malignant bone tumor that arises from mesenchymal cells, which are also the source of MSCs. While MSCs play a role in bone regeneration, they also influence the tumor microenvironment in osteosarcoma. Metallothionein 1G (MT1G) regulates the cell’s antioxidant status and metal ion balance, influencing the proliferation and differentiation of MSCs, thereby affecting bone regeneration and repair processes, MT1G has been verified as a target for osteosarcoma (55). In addition, MSC-derived exosomes as targeted nanocarriers for Doxorubicin delivery, enhancing osteosarcoma therapy through the SDF1-CXCR4 axis (56). However, challenges remain in optimizing MSC therapy, including issues related to cell survival, engraftment, and long-term efficacy.

Allogeneic MSC transplantation offers several advantages, making it an attractive option for regenerative therapies (57, 58). One key benefit is the off-the-shelf availability of allogeneic MSCs, which eliminates the delays and complications associated with autologous MSC sourcing. Since these cells can be readily harvested from a donor, the need for patient-specific cell harvesting is bypassed, facilitating more timely treatments (58). In addition, allogeneic MSCs have immunomodulatory properties that enable them to modulate the immune response, which is crucial for reducing inflammation and promoting healing in various tissues. These properties allow them to be effective in treating diseases characterized by chronic inflammation, such as osteoarthritis, by helping to suppress immune-mediated tissue damage.

Furthermore, fetal-derived MSCs have garnered attention due to their higher proliferative capacity and enhanced differentiation potential compared to adult-derived MSCs (59). These cells are known to proliferate more rapidly and have a greater ability to differentiate into a wider range of cell types. As a result, fetal-derived MSCs present a promising alternative source for regenerative medicine, particularly in the context of orthopedic diseases where rapid tissue regeneration is essential (60). Their superior growth and differentiation potential could lead to more effective MSC-based therapies, improving the outcomes for patients with conditions such as osteoarthritis or bone fractures.

### 2.2 Chondrocyte-based cell therapy

Chondrocyte-based therapies involve the transplantation of autologous or allogenic chondrocytes to repair damaged cartilage (61, 62). It has been widely used in the treatment of focal cartilage defects, particularly in younger patients (63). The process typically involves harvesting healthy chondrocytes from non-weight-bearing regions of the joint, expanding them *in vitro*, and then re-implanting them into the damaged area, thus to restore the normal cartilage structure and function, thereby alleviating symptoms and preventing further joint degeneration (64). Autologous chondrocyte implantation (ACI) is a well-established procedure for treating large cartilage defects and has been shown to improve joint function and reduce pain in many patients (65). The key advantage of ACI is the use of the patient’s own cells, which minimizes the risk of immune rejection (66). Both ACI and matrix-assisted chondrocyte implantation (MACI) procedures have demonstrated long-term success in treating knee cartilage lesions related to osteoarthritis, helping to delay the need for arthroplasty (66).

However, technique has limitations, such as the need for a two-step procedure and the potential for donor site morbidity. Additionally, the newly generated cartilage may not fully replicate the mechanical properties of native cartilage, leading to a potential risk of graft failure or degeneration over time. Using scaffold materials combined with chondrocytes to provide structural support and promote the growth of new cartilage (67). These scaffolds are made from a variety of materials, including natural polymers, synthetic hydrogels, or even decellularized tissues, and they are designed to mimic the biomechanical properties of cartilage (68–70). Silk fibroin-based materials have been used for cartilage and osteochondral repair too (71). Advances in 3D bioprinting have enabled the creation of more complex scaffolds that better support chondrocyte growth and tissue regeneration (72). Additionally, the use of stroma/stem cells, such as MSCs or iPSCs, in combination with chondrocytes or scaffolds may offer a promising strategy to enhance cartilage regeneration and improve long-term outcomes.

A significant challenge in cartilage therapies is the *in vitro* culture and expansion of autologous chondrocytes. During culture, chondrocytes can dedifferentiate, losing their ability to produce cartilage-specific extracellular matrix components (73). Moreover, the need to expand sufficient numbers of cells for clinical applications remains a challenge, as does maintaining the differentiated state of the chondrocytes. One key question regarding autologous chondrocyte transplantation is whether it leads to true regeneration of articular cartilage or primarily facilitates tissue repair. While some studies report the formation of hyaline-like cartilage, others suggest that the newly formed tissue may more closely resemble fibrocartilage, which lacks the durability and biomechanical properties of genuine hyaline cartilage.

### 2.3 Engineered immune cell therapy

Engineered immune cells, such as chimeric antigen receptor T cells (CAR-T), CAR-natural killer (CAR-NK) cells, and CAR-macrophages (CAR-M), represent an exciting frontier in cell-based therapies. Traditionally used in cancer immunotherapy, these engineered immune cells have also shown potential for modulating inflammation and promoting tissue repair in orthopedic diseases. They focus on harnessing the immune system’s ability to target and eliminate specific cells, such as inflammatory macrophages or damaged tissue, and to enhance tissue repair processes.

Engineered immune cell therapy has been widely studied in orthopedic cancers such as osteosarcoma, chondrosarcoma, Ewing’s sarcoma, fibrosarcoma, etc (74, 75). Phase I trial (NCT02107963) of GD2 CAR-Ts (GD2-CAR.OX40.28.z.iC9), has demonstrated feasibility and safety of administration in children and young adults with osteosarcoma and neuroblastoma (76). GGD2-C7R CAR-T therapy clinical trial (NCT03635632) are conducted in solid tumors with GD2 target including relapsed Ewing Sarcoma and osteosarcoma (77). These trials highlight the promising potential of engineered immune cell therapies to treat difficult-to-treat orthopedic cancers.

The ability of CAR-T cells to specifically target inflammatory cells, such as activated macrophages or T cells, could be utilized to reduce inflammation and promote tissue healing in diseases like osteoarthritis. Additionally, CAR-T cells could be engineered to target specific molecules involved in cartilage degradation or bone resorption, such as matrix metalloproteinases (MMPs) or receptor activator of nuclear factor kappa-B ligand (RANKL). CAR-NK cells are engineered to enhance the cytotoxic activity of NK cells, and their application in orthopedic diseases could involve targeting and eliminating activated immune cells that contribute to chronic inflammation. Additionally, CAR-NK cells may promote tissue regeneration by enhancing the activity of other immune cells, such as MSCs, within the tissue microenvironment (78). In addition, macrophages play a dual role in inflammation and tissue repair. Macrophages could be polarized *in vitro* in two extreme phenotypes: the pro-inflammatory (M1-like) phenotype, associated with the release of pro-inflammatory cytokines and promotion of tissue destruction, and the anti-inflammatory (M2-like) phenotype, which supports tissue regeneration and repair (79). During the resolution phase of inflammation, pro-resolving macrophages play a crucial role in promoting tissue healing by producing growth factors such as vascular endothelial growth factor (VEGF) and platelet-derived growth factor (PDGF), which enhance angiogenesis and collagen synthesis (80, 81). Given their ability to modulate immune responses, CAR-macrophages could be explored as a potential approach to attenuate inflammation and support tissue regeneration in orthopedic diseases. Future studies may investigate their capacity to target pro-inflammatory cytokines or signaling pathways involved in cartilage degradation.

### 2.4 Induced pluripotent stem cells therapy

IPSCs offer an abundant and renewable source of cells for tissue engineering, serving as an appealing alternative to primary cells. With their remarkable plasticity and differentiation capabilities, iPSCs hold significant promise for cell-based therapies. Patient-specific iPSCs can be tailored to reduce the risk of autoimmune reactions, making them a nearly ideal candidate for regenerative medicine. Research on iPSCs in cartilage tissue engineering has highlighted their potential for functional cartilage repair and as valuable models for understanding cartilage-related diseases. For instance, iPSCs derived from somatic cells generate patient-specific stem cells for studying osteoarthritis and testing therapeutic agents (82, 83). Furthermore, iPSCs obtained from OA patients’ tissues can differentiate into cartilage, creating new opportunities for investigating cartilage pathology and treatment (84). Despite these advancements, no clinical trials using iPSC-derived cartilage cells for therapy have been reported. iPSCs share the proliferative and differentiation advantages of other stem cells while avoiding issues related to immune rejection and ethical concerns (24). However, further research is essential to advance the use of iPSC-derived chondrocytes in OA treatment and joint repair. In addition to cartilage repair, iPSC-based therapies are showing potential in the treatment of bone-related disorders. For example, iPSCs have been successfully differentiated into osteoblasts, the cells responsible for bone formation, and used in preclinical studies to promote bone regeneration in critical-sized bone defects. In a study involving a mouse model, iPSC-derived osteoblasts seeded onto bioengineered scaffolds not only enhanced bone regeneration but also demonstrated superior integration with the host tissue compared to traditional stem cell therapies (85). This approach highlights the versatility of iPSCs in addressing complex orthopedic challenges such as large bone defects and non-union fractures.

As outlined in the previous sections, a range of cell-based therapies has been developed to address orthopedic diseases, each offering distinct mechanisms and therapeutic potentials. To fully assess the advantages and limitations of these approaches, it is essential to conduct a comparative analysis of several key therapies, including MSC therapy, chondrocyte therapy, immune cell therapy (specifically CAR-T cell therapy for osteosarcoma), and iPSC therapy. This comparison enables a comprehensive evaluation of their effectiveness in promoting tissue regeneration, targeting tumors, and achieving optimal therapeutic outcomes (**Table 2**).

**Table 2:** Comparison of different cell therapies in orthopedic diseases.

Aspect	Mesenchymal stem cell therapy	Chondrocyte therapy	Immune cell therapy	Induced pluripotent stem cell therapy
Source of cells	Bone marrow, adipose tissue, umbilical cord, etc.	Cartilage tissue (autologous or allogenic)	Immune cells (e.g., T cells, macrophages)	Patient-derived somatic cells reprogrammed into iPSCs
Differentiation potential	Multipotent, can differentiate into cartilage, bone, or adipose tissue	Already differentiated as chondrocytes	No differentiation; directly target immune modulation	Pluripotent, can differentiate into any cell type, including chondrocytes
Primary mechanism	Regeneration and repair through differentiation and paracrine signaling	Directly regenerate cartilage tissue	Modulate inflammation and promote repair	Generate functional cells for transplantation
Target diseases	Osteoarthritis, bone defects, cartilage injuries	Cartilage injuries, osteochondral defects	Autoimmune-related orthopedic diseases, inflammation-associated injuries	Osteoarthritis, cartilage and bone regeneration
Clinical use	Widely studied, some therapies in clinical trials or approved	Established in clinical use (e.g., ACI)	Emerging, limited to preclinical/early trials	Experimental, primarily in preclinical research
Advantages	Easy to harvest, immunomodulatory properties, no tumorigenesis risk	Highly specific to cartilage repair, clinically established methods	Targets immune-driven inflammation and TME modulation	High differentiation potential, can generate unlimited cell supply
Challenges	Limited differentiation efficiency, poor survival in hostile environments	Limited proliferation potential, donor site morbidity	High cost, off-target effects, complex engineering	Tumorigenesis risk, high cost, ethical concerns
Regulatory status	Some approved therapies; others under evaluation	Well-established clinical guidelines	Preclinical and early-phase trials	Experimental, facing strict regulatory scrutiny

## 3 Immune modulation in orthopedic repair and regeneration

### 3.1 Balancing inflammation and regeneration

The immune microenvironment plays a crucial role in bone and cartilage repair and recovery following orthopedic tumor treatments (86, 87). Effective healing requires a well-regulated immune response to clear damaged cells and pathogens while promoting tissue regeneration. Acute inflammation initiates repair in the early stages of injury, but excessive or chronic inflammation can hinder recovery, leading to cartilage degradation, fibrosis, impaired bone healing, and delayed recovery after tumor surgery (88). Achieving a balance between inflammation and regeneration is critical in orthopedic conditions like osteoarthritis, bone fractures, cartilage degeneration, and tumor-related treatments. Key immune mediators, including cytokines and immune cells, influence this balance. Pro-inflammatory cytokines like TNF-α, IL-1β, and IL-6 are essential for initiating repair but can exacerbate tissue damage if persistently elevated (89). In contrast, anti-inflammatory cytokines such as IL-10 and TGF-β resolve inflammation and support tissue healing by promoting cell migration and differentiation. In orthopedic tumors, regulatory T cells (Tregs) suppress excessive inflammation, creating a regenerative microenvironment conducive to tissue healing after surgery (90). Modulating the immune response is therefore a promising strategy to enhance tissue repair and improve cell therapy outcomes.

### 3.2 The role of TLR3 in immune modulation

TLR3, a pattern recognition receptor, is critical for modulating inflammation and repair (91). Expressed on immune cells, chondrocytes, and synovial cells, TLR3 detects double-stranded RNA from pathogens or tissue damage (92, 93). Its activation triggers NF-κB and IRF3 signaling, leading to the production of pro-inflammatory cytokines and type I interferons. Activating TLR3 selectively in MSCs enhances their regenerative properties, improving integration and functionality in damaged tissues, thus promote joint degeneration in osteoarthritis (34).While TLR3-induced inflammation supports innate immunity, it also impacts tissue homeostasis and repair. In orthopedic diseases, TLR3 signaling plays a dual role. It contributes to osteoarthritis by promoting pro-inflammatory cytokine production, exacerbating cartilage degradation and inflammation. Targeting TLR3 by alleviate osteoarthritis (19). However, TLR3 also enhances the regenerative potential of MSCs, improving their migration and differentiation. Preconditioning MSCs with TLR3 agonists boosts chondrogenic differentiation and migration, which enhances cartilage repair. This dual role makes TLR3 an attractive target for therapeutic modulation.

## 4 Challenges and potential strategies of cell therapy for orthopedic diseases

Key challenges in cell therapy for orthopedic diseases include improving cell survival, enhancing engraftment within damaged tissues, and mitigating immunological and inflammatory responses that impair tissue repair, particularly in the complex tumor microenvironment of orthopedic cancers (94). Additionally, the lack of standardized protocols for cell production, expansion, and administration poses significant hurdles to ensuring consistency and reproducibility. Variability in cell sources, techniques, and delivery methods further complicates the translation of these therapies into clinical practice. Regulatory barriers remain another obstacle, with approval processes for cell-based therapies being both time-consuming and demanding due to stringent safety and efficacy requirements. To address these challenges, emerging technologies such as advanced biomaterials, nanocarriers, and bioengineering tools are being explored. Biomaterials like 3D-printed scaffolds, injectable hydrogels, and electrospun fibers provide mechanical support for transplanted cells while mimicking the natural extracellular matrix to promote cellular adhesion, proliferation, and differentiation (95–97). Nanotechnology further supports cell therapy by enabling targeted delivery of therapeutic agents or enhancing the bioactivity of transplanted cells. Nanoparticles and nanofibers encapsulate and deliver drugs, genes, or signaling molecules directly to the injury site, reducing off-target effects and improving therapeutic outcomes (98). For example, nanocarriers loaded with anti-inflammatory cytokines or immunosuppressive agents modulate the immune response to improve engraftment and tissue repair in inflammatory environments (99). Furthermore, preconditioning strategies are also emerging as a solution to improve cell survival and functionality. Exposing cells to hypoxic conditions, pro-inflammatory cytokines, or specific signaling molecules prior to transplantation can enhance their resilience in hostile microenvironments and increase their regenerative potential (100). Moreover, genetic engineering of cells, such as modifying MSCs to express anti-apoptotic or pro-regenerative factors, is being explored to further boost their therapeutic efficacy. Successful regenerative therapy for articular cartilage must achieve specific cellular and morphological characteristics, including the restoration of a well-organized cartilage structure, proper integration with subchondral bone, and the formation of extracellular matrix (ECM) components like collagen type II and proteoglycans. These features are essential for the functionality and durability of the regenerated cartilage in joint repair.

## 5 Conclusions and perspectives

In summary, cell-based therapies have made significant strides in the treatment of orthopedic diseases, offering potential solutions for conditions such as osteoarthritis, cartilage degeneration, bone fractures, and orthopedic tumors like osteosarcoma, chondrosarcoma, and Ewing sarcoma. These therapies promote tissue repair, restore function, and reduce inflammation, providing a promising alternative to conventional treatments that primarily manage symptoms. MSCs, chondrocyte transplantation, and engineered immune cell therapies have shown great potential in preclinical studies and early clinical trials. Despite these advancements, several challenges remain in translating these therapies into widespread clinical use.

Looking forward, a multi-faceted approach combining advanced cell therapies with immune modulation, molecular targeting, and emerging technologies is essential to improving clinical outcomes in orthopedic diseases and tumors. However, several unresolved questions remain that need to be addressed in future research:

1. How can we improve cell survival after transplantation? Ensuring the survival and function of transplanted cells, especially in hostile environments like the tumor microenvironment, is challenging.
2. How can we balance inflammation and regeneration? Inflammation is crucial for repair but excessive immune responses hinder regeneration.
3. How can we scale up cell therapies for clinical use? Standardized protocols for producing and administering cell therapies are lacking.
4. What molecular pathways can enhance regeneration? Pathways like TLR3 are linked to inflammation and repair, but their roles are not fully understood.

In conclusion, while cell-based therapies hold immense promise for the treatment of orthopedic diseases and tumors, overcoming the remaining challenges will require a multidisciplinary approach. The integration of immune modulation, advanced technologies, and a deeper understanding of molecular pathways will be key to enhancing the efficacy of these therapies and achieving better clinical outcomes. The future of orthopedic disease and tumor treatment will likely involve a personalized, tailored approach, combining the best of cell therapy, immune modulation, and emerging technologies to provide optimal solutions for patients.
